# Genome Degeneration and Adaptation in a Nascent Stage of Symbiosis

**DOI:** 10.1093/gbe/evt210

**Published:** 2014-01-08

**Authors:** Kelly F. Oakeson, Rosario Gil, Adam L. Clayton, Diane M. Dunn, Andrew C. von Niederhausern, Cindy Hamil, Alex Aoyagi, Brett Duval, Amanda Baca, Francisco J. Silva, Agnès Vallier, D. Grant Jackson, Amparo Latorre, Robert B. Weiss, Abdelaziz Heddi, Andrés Moya, Colin Dale

**Affiliations:** ^1^Department of Biology, University of Utah; ^2^Institut Cavanilles de Biodiversitat i Biologia Evolutiva, Universitat de València, Spain; ^3^Department of Human Genetics, University of Utah; ^4^INSA-Lyon, INRA, UMR203 BF2I, Biologie Fonctionnelle Insectes et Interactions, Villeurbanne, France; ^5^Área de Genómica y Salud, Fundación para el Fomento de la Investigación Sanitaria y Biomédica de la Comunitat Valenciana FISABIO – Salud Pública, Valencia, Spain

**Keywords:** recent symbiont, degenerative genome evolution, IS elements, pseudogenes, comparative genomics

## Abstract

Symbiotic associations between animals and microbes are ubiquitous in nature, with an estimated 15% of all insect species harboring intracellular bacterial symbionts. Most bacterial symbionts share many genomic features including small genomes, nucleotide composition bias, high coding density, and a paucity of mobile DNA, consistent with long-term host association. In this study, we focus on the early stages of genome degeneration in a recently derived insect-bacterial mutualistic intracellular association. We present the complete genome sequence and annotation of *Sitophilus oryzae* primary endosymbiont (SOPE). We also present the finished genome sequence and annotation of strain HS, a close free-living relative of SOPE and other insect symbionts of the *Sodalis*-allied clade, whose gene inventory is expected to closely resemble the putative ancestor of this group. Structural, functional, and evolutionary analyses indicate that SOPE has undergone extensive adaptation toward an insect-associated lifestyle in a very short time period. The genome of SOPE is large in size when compared with many ancient bacterial symbionts; however, almost half of the protein-coding genes in SOPE are pseudogenes. There is also evidence for relaxed selection on the remaining intact protein-coding genes. Comparative analyses of the whole-genome sequence of strain HS and SOPE highlight numerous genomic rearrangements, duplications, and deletions facilitated by a recent expansion of insertions sequence elements, some of which appear to have catalyzed adaptive changes. Functional metabolic predictions suggest that SOPE has lost the ability to synthesize several essential amino acids and vitamins. Analyses of the bacterial cell envelope and genes encoding secretion systems suggest that these structures and elements have become simplified in the transition to a mutualistic association.

## Introduction

Intracellular mutualistic bacteria are notable among cellular life forms because they maintain extremely small genomes. Many examples exist (Nakabachi et al. 2006; Pérez-Brocal et al. 2006; McCutcheon and Moran 2007, 2010; McCutcheon et al. 2009) and the smallest is currently the symbiont of the phloem-feeding insect pest *Macrosteles quadrilineatus,* “*Candidatus* Nasuia deltocephalinicola,” with a 112 kb genome encoding just 137 protein-coding genes (Bennett and Moran 2013). Such small genomes are derived from a degenerative process that is predicted to take place over several hundred million years and is accompanied by an increased rate of DNA and polypeptide sequence evolution (Pérez-Brocal et al. 2006), and often a dramatic nucleotide composition bias that results in an increased ratio of adenine and thymine residues (Andersson JO and Andersson SGE 1999). Because endosymbiotic bacteria are isolated inside specialized cells (bacteriocytes) within their host, opportunities to engage in parasexual genetic exchange are greatly reduced in comparison to free-living bacteria. The resulting evolutionary trajectory is therefore characterized by irreversible gene inactivation and loss; a process that is predicted to be accelerated by a reduced efficiency of selection resulting from frequent population bottlenecks that reduce the effective population size (*N*_e_) during host reproduction (Moran 1996; Mira et al. 2001; Silva et al. 2003; Schmitz-Esser et al. 2011).

Although many highly reduced endosymbiont genomes have now been sequenced and analyzed, little research has focused on recently derived examples and the forces shaping genome evolution in the early stages of an endosymbiotic association. To address this issue, we conducted a comparative analysis of the genome sequences of two recently derived insect symbionts, *Sitophilus oryzae* primary endosymbiont (SOPE) and *Sodalis glossinidius* (a secondary symbiont of tsetse flies) and a closely related free-living bacterium, designated “strain HS” (Clayton et al. 2012). The characterization of strain HS and related *Sodalis*-allied insect symbionts revealed that genome degeneration is extremely potent in the early stages of a symbiotic association. In the case of SOPE, genome degeneration catalyzed the loss of over 50% of the symbiont gene inventory in a very short period of time (Clayton et al. 2012).

Strain HS was discovered as a novel human-infective bacterium, isolated from a hand wound following impalement with a tree branch. Phylogenetic analyses placed strain HS on a clade comprising the *Sodalis*-allied insect endosymbionts, including SOPE and *So. glossinidius*. Preliminary genomic comparative analyses of the gene inventories of strain HS, SOPE, and *So. glossinidius* were compatible with the notion that strain HS has a gene inventory resembling a free-living common ancestor that has given rise to mutualistic bacterial symbionts in a wide range of insect hosts (Clayton et al. 2012).

In this study, we report the complete genome sequence and annotation of both SOPE and strain HS. We also propose the formal nomenclature *Candidatus* Sodalis pierantonius str. SOPE to replace the more commonly used name SOPE. Although SOPE shares characteristics with ancient obligate intracellular symbionts, including strict maternal inheritance, residence in bacteriocytes, and nutrient provisioning, it has a relatively large genome with many pseudogenes and mobile genetic elements, consistent with the notion that it is a recently derived symbiont. We describe the predicted metabolic capabilities of SOPE and explain how an expansion of insertion sequence (IS) elements has mediated large-scale genomic rearrangements, some of which may be adaptive in nature. Further comparisons between the genomes of SOPE, *So. glossinidius,* and strain HS shed light on the adaptive changes taking place early in the evolution of insect symbionts.

## Materials and Methods

### SOPE Shotgun Library Construction and Sequencing

Shotgun library construction and sequencing was performed as described by Clayton et al. (2012), briefly, 60 µg of genomic DNA was sheared to a mean fragment size of 10 kb, end repaired, and adaptors were blunt-end ligated to the fragments. Fragments in the size range of 9.5–11.5 kb were gel purified after separation in a 1% agarose gel. Fragments were ligated into a plasmid vector and transformed into chemically competent *Escherichia coli* cells. Runaway plasmid replication was induced, and plasmid DNA was purified by alkaline lysis, and cycle sequencing reactions were performed. The reactions were ethanol precipitated, resuspended, and then sequenced on an ABI capillary sequencer.

### SOPE Genome Sequence Assembly, Finishing, and Validation

Genome sequence assembly, finishing, and validation were performed as described by Clayton et al. (2012). Filtered reads were assembled using the Phusion assembler (Mullikin and Ning 2003), and after inspection of the initial contigs, gaps were closed using a combination of iterative primer walking and gamma-delta transposon-mediated full-insert sequencing of plasmid clones. Validation was performed by mapping 1,404 paired-end sequence reads generated from a SOPE fosmid library on to the finished genome assembly.

### SOPE Genome Annotation

The assembled genome sequence of SOPE was submitted to the National Center for Biotechnology Information (NCBI) Prokaryotic Genomes Automatic Annotation Pipeline (PGAAP) for annotation. The resulting candidate open reading frames (ORFs) were then aligned to the HAMAP database (Lima et al. 2009) and classified according to their percent protein identity and length. ORFs that had more than 90% protein identity and more than 80% of the length of the database match and did not contain frameshifts or premature stop codons were classified as intact ORFs. The remaining candidate ORFs were then classified as intact or pseudogenes by generating a Blast database from the top HAMAP result for each candidate ORF, then two nucleotide query files were generated: one based on the PGAAP annotation and another including 2,500 nucleotides on either end of the candidate ORF. BlastX searches against the database generated from the top HAMAP result were then performed on each query file, and the output was parsed to search for extended protein matches that indicated either truncated candidate ORFs or possible frameshifted candidate ORFs. The annotation was then aligned to the draft genome sequence and annotation of strain HS using the Smith-Waterman algorithm implemented in cross_match (Gordon et al. 1998). Custom Perl scripts were used to identify any ORFs not identified by PGAAP as well as refine and classify ORFs as intact or pseudogenized. ORFs not identified by PGAPP but spanning more than 99% of the orthologous strain HS ORF or more than 90% of ORFs smaller than 300 nucleotides in size were annotated as intact only if no inactivating mutation were present. Additional manual curation was preformed with extensive use of the Bacterial Annotation System (BASys) (van Domselaar et al. 2005) and EcoCyc databases (Keseler et al. 2013). IS elements were identified and annotated using ISSaga (Varani et al. 2011). The resulting annotation was also manually curated and adjusted in Artemis (Rutherford et al. 2000).

### Strain HS Genome Sequence Finishing and Annotation

The strain HS draft genome sequence and annotation was generated as described previously (Clayton et al. 2012). To close the genome sequence of strain HS, 16.5 µg of genomic DNA was submitted to Macrogen Inc. (Macrogen, Inc. Seoul, South Korea) for sequencing on the Roche 454 GS-FLX (454 Life Sciences, a Roche company. Branford, CT) platform. A total of 804,816 (543,754 paired-end) reads were generated from a 5-kb insert size mate pair library. These reads along with 34 million paired-end Illumina reads of 55 bases in length were assembled with Newbler 2.7 (Margulies et al. 2005). The resulting assembly consisted of two scaffolds containing 47 contigs covering 5.1 Mbp. These gaps were then closed computationally (by incorporating gap filling reads) or by Sanger sequencing of polymerase chain reaction products derived from gaps yielding a closed circular chromosome of 4.7 Mbp and a circular megaplasmid of 449.8 kb.

### 16S rRNA Mutation Analysis

Sequence alignments were generated using MUSCLE (Edgar 2004) for the 16S rRNA genes from strain HS, SOPE, SZPE, and *So. glossinidius*. PhyML (Guindon et al. 2010) was then used to construct a phylogenetic tree using the HKY85 (Hasegawa et al. 1985) model of sequence evolution with 25 random starting trees and 100 bootstrap replicates. Classification of mutations in the 16S rRNA stem regions was preformed as described by Pei et al. (2010) and classification of mutations in the entire 16S rRNA sequence was preformed as described by Wuyts (2001).

### Nucleotide Substitution Rates and Predicted Cryptic Pseudogenes

Orthologous genes in SOPE, *So. glossinidius,* and strain HS were determined using OrthoMCL (Li 2003) with recommended parameters (Fischer et al. 2002). Before input into OrthoMCL all pseudogenes, IS elements, and phage sequences were removed from the sets of SOPE and *So. glossinidius* genes. The output of OrthoMCL was then screened, and any nonorthologous genes or low-quality matches were discarded, and a total of 1,601 strain HS orthologous genes were obtained for SOPE and 1,734 for *So. glossinidius*. Sequence alignments for each orthologous gene pair was generated using MUSCLE (Edgar 2004). Pairwise estimates of the synonymous (d*S*) and nonsynonymous (d*N*) substitution rates were obtained from the YN00 program of the PAML 4.6 package (Yang 2007). The Processing Development Environment (www.processing.org, last accessed January 3, 2014) was used to plot d*N* and d*S* for each strain HS–SOPE pairwise comparison and to compute mean ORF sizes. A plot was also generated to compare the d*N*/d*S* values of all intact orthologs maintained by SOPE and *So. glossinidius*.

### Functional Analysis

The Artemis Comparison Tool (Carver et al. 2005) was used to perform a pairwise comparison between the genomes of SOPE, strain HS, and *So. glossinidius*, to explore conservation of synteny, and to help in the identification of orthologous genes and pseudogenes, to identify similarities and discrepancies in the functional capabilities of these organisms. The reannotated genome of *So. glossinidius* was used in this comparison (Belda et al. 2010). Metabolic capabilities were analyzed with Blast2Go (Conesa et al. 2005) and KAAS (Moriya et al. 2007) programs and manually curated. Functional information was retrieved from the BioCyc (Caspi et al. 2010), KEGG (Ogata et al. 1999), and BRENDA (Scheer et al. 2011) databases and extensive literature searches.

### Genomic Rearrangements Between SOPE and Strain HS

To identify all genomic rearrangements between SOPE and strain HS, we performed a fully recursive search of the SOPE genome using all 20-mers derived from the complete strain HS genome sequence. Both the search and subsequent data plotting were performed in the Processing Development Environment (www.processing.org, last accessed January 3, 2014). The consensus FtsK orienting polar sequences (KOPS) site used for the plot in figure 2 was GGGNAGGG and the *dif* (the chromosomal site where the XerCD recombinase decatenates and resolves chromosome dimers) site is AGTACGCATAATACATATTATGTTAAAT.

### Rendering Genomic Features

Scalar diagrams of 1) two chromosomal clusters containing large numbers of IS elements and 2) regions encoding type III secretion systems (TTSS) in *So. glossinidius*, SOPE, and strain HS were rendered in the Processing Development Environment (www.processing.org, last accessed January 3, 2014).

### Data Availability

The *Candidatus* Sodalis pierantonius str. SOPE genome sequence and annotation was deposited in GenBank under the accession number CP006568. The strain HS chromosome and plasmid sequence and annotation were deposited in GenBank under the accession numbers CP006569 and CP006570, respectively.

## Results

### General Features of the SOPE and Strain HS Genome Sequences

**Fig. 1.—:**
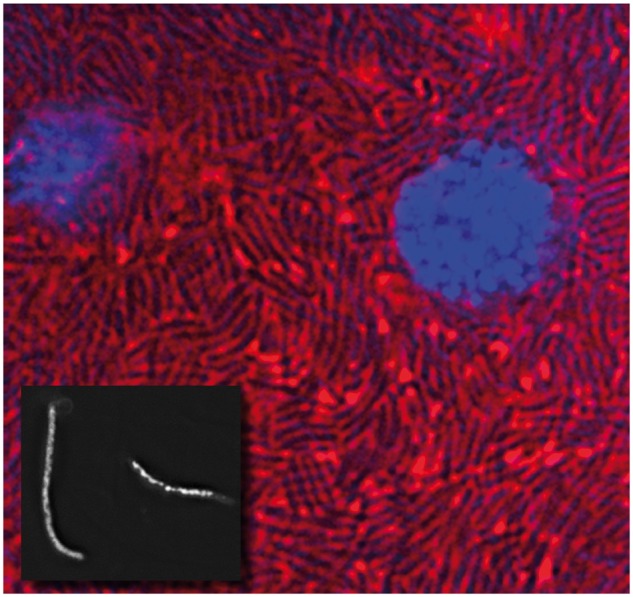
Microscopic images of SOPE. The main panel shows cells from a 5th instar bacteriome of SOPE stained with FM4-64 (red) and DAPI (blue). Rod-shaped bacteria are densely packed into the cytoplasm of the insect cells, whose nuclei display extensive DAPI staining. The inset panel shows isolated SOPE cells stained with DAPI, illustrating their filamentous morphology.

SOPE is an intracellular, bacteriome-associated symbiont that resides in host bacteriocytes (fig. 1) that has a genome consisting of one circular chromosome of 4,513,140 bases with an average GC content of 56.06%. A total of 4,080 candidate protein-coding sequences (CDSs) were annotated of which 2,309 (56.6%) are predicted to be intact based on the absence of frame shift mutations, premature stop codons, or truncating deletions, whereas 1,771 (43.4%) candidate CDSs are predicted to be pseudogenes maintaining one or more these mutations. Mutations were identified by aligning 2,731 homologous CDSs shared between SOPE and strain HS, excluding mobile genetic elements such as integrated prophage islands and IS elements. Since the gene inventory of SOPE is known to be a subset of strain HS, and the sequences of strain HS and SOPE are very closely related (having only ∼2% synonymous divergence genome wide [Clayton et al. 2012]), the genome sequence of strain HS provided a unique opportunity to accurately identify all the mutations leading to predicted ORF inactivations in SOPE.

The complete genome sequence of strain HS consists of a circular chromosome of 4,709,528 bases and one mega plasmid of 449,897 bases. The average GC content of the chromosome is 57.47%, and the plasmid GC content is 53.22%. There are a total of 3,993 CDSs encoded on the chromosome with only 61 pseudogenes and 365 CDSs encoded on the mega-plasmid with 14 predicted pseudogenes. Pseudogenes in strain HS were identified based on alignments of the CDSs with homologs from closely related bacteria in the NCBI database.

### An Epidemic of IS Element Expansion in SOPE

The genome of SOPE is notable because it has undergone a massive expansion of bacterial IS elements, which accounts for a total of 0.83 Mbp (18%) of the chromosome. The genome contains a total of 822 CDSs encoding either transposases or helpers of transposition that are encoded within 804 IS elements. This expansion consists of four major IS elements, ISSoEn 1 to 4 (previously described as ISsope1 to 4) (Gil Garcia et al. 2008), belonging to the IS families named IS5 (ssgr IS903), IS256, IS21, and ISL3, respectively. These four families constitute 795 of the 804 total IS element CDSs in the genome. Within each family, the percent nucleotide identity was greater than 94%, indicating recent expansion within the SOPE chromosome, or maintenance of a high level of sequence identity through gene conversion. Table 1 summarizes the number of intact and disrupted copies of each of the main IS types. The remaining nine IS elements consist of six copies of the ISPlu15 family, one copy of the IS418 family, and five copies of a Mu-like transposase.

**Table 1:** Summary of the Four Major IS Elements in the SOPE Genome

	ISSoEn1	ISSoEn2	ISSoEn3 (Transposase)	ISSoEn3 (Helper of Transposition)	ISSoEn4
IS family	IS5 (ssgr IS903)	IS256	IS21	IS21	ISL3
IS elements with intact ORFs	189	104	49	61	9
IS elements with disrupted ORFs	122	160	68	38	10
Total	311	264	117	99	19

**Fig. 2.—:**
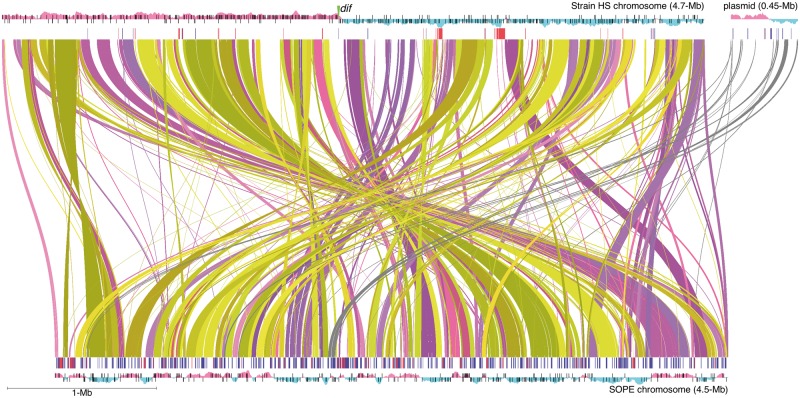
Whole-genome sequence alignment of strain HS and SOPE. Bezier curves highlight regions of synteny shared between strain HS and SOPE. Uninterrupted blocks of synteny are rendered in the same hue. Matches occurring on the same DNA strand are rendered in the purple spectrum, whereas those occurring on different strands are rendered in the yellow spectrum. Thus, to maintain replicational symmetry, matches highlighted in purple should remain on the same replichore, whereas those highlighted in yellow should switch replichore. Sequences shared between the strain HS megaplasmid and SOPE chromosome are rendered in gray. The outer plots represent GC skew with positive skew depicted in red and negative skew depicted in blue. The strand-specific locations of KOPS (FtsK orienting polar sequences) sites are shown as tickmarks overlaid on the plot of GC skew. Inside the plots, the pink and blue tick marks correspond to the positions of phage and IS element sequences, respectively. These and any other forms of repetitive DNA were masked in the generation of the alignment. The location of the *dif* (terminus) sequence in strain HS is highlighted and intersects with the switch in GC skew, as expected.

**Fig. 3.—:**
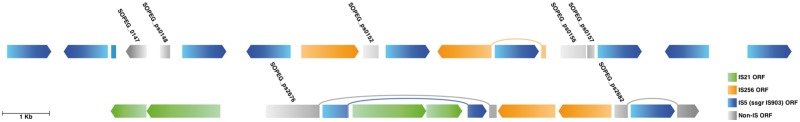
IS element dense regions in SOPE. Scalar illustration of two IS element dense regions in the SOPE chromosome. The top row corresponds to the region encompassing SOPEG_ps0144-SOPEG_0160, and the bottom row corresponds to SOPEG_2674-SOPEG_2683. IS element ORFs are colored according to their family (see key). ORFs are shaded in accordance with their positional synteny in comparison with a full length HS ortholog. The full spectrum of shading (5′–3′) is depicted in the key. ORFs disrupted by an IS element insertion are connected by curved lines. All non-IS element ORFs are labeled with their SOPE locus tags.

Extensive expansions of IS elements have been documented in a number of bacteria undergoing lifestyle transitions (Parkhill et al. 2003; Moran and Plague 2004), implying that they are a typical component of the process of degenerative evolution. Indeed, it has been proposed that such IS element proliferations take place as a consequence of the imposition of relaxed selection on a large number of genes (Moran and Plague 2004; Plague et al. 2008), facilitating the expansion of IS elements into genomic space encompassing genes evolving under relaxed selection. However, in the case of SOPE, only a small proportion of IS elements were found to occupy genic sequences with the majority of these elements clustering in intergenic regions (fig. 2). Despite the high level of nucleotide identity within IS families, the sequence strategy facilitated assembly of chromosomal regions harboring both dense IS clusters and genome duplications (Clayton et al. 2012). Examples of two IS-dense intergenic clusters in the SOPE genome are depicted in figure 3. We previously rationalized the clustering of IS elements on the basis that it might be favored by natural selection to avoid the interruption of vital genic sequences (Clayton et al. 2012). At face value, this appears to contradict the notion that IS element expansions occur as a direct consequence of the emergence of neutral space. However, the expansion of IS elements into just a small proportion of neutral space might be sufficient to precipitate an epidemic of activity, as a simple consequence of increasing IS element copy number. Indeed, it has been shown that IS element transposases have the capability to act in trans (Derbyshire et al. 1990; Derbyshire and Grindley 1996), such that a transposase derived from one element could catalyze the transposition of other elements in the genome. To this end it is interesting to note that many of the transposase genes in the IS elements of SOPE are pseudogenized. Although this could be taken as a sign that the epidemic of transposition is waning, it is also conceivable that those IS elements with inactive transposases are still being mobilized in trans.

Similar to many nonhost-associated bacteria, strain HS has very few IS elements (Wagner et al. 2007). The existing predicted transposase CDSs are either small fragments of IS elements or ORFs that have been disrupted by inactivating mutations. Strain HS and SOPE contain homologs of the ISNCY ssgr ISPlu15 family IS element; however, this element only accounts for a very small fraction of the high number of IS elements in the SOPE genome. Of the four major IS element families in SOPE, none are found in the strain HS genome, but *So. glossinidius* does maintain the IS element belonging to the IS5 family (named ISSgl1 in this species; Belda et al. 2010), which is the most abundant in SOPE. Thus, the IS5 element may have been present in the last common ancestor of all three bacteria and then subsequently lost in strain HS, or it is also possible that SOPE and *So. glossinidius* independently acquired this element.

### Evidence for Extensive Recent Intragenomic Rearrangements in SOPE

Although strain HS and SOPE share a very high level of sequence identity, consistent with the notion of recent common ancestry (Clayton et al. 2012), a genome wide alignment of homologous sequences in strain HS and SOPE revealed a surprisingly low level of genome-wide synteny (fig. 2). Although this lack of synteny could be explained as a consequence of rearrangements in either lineage, there are two compelling lines of evidence indicating that the rearrangements have predominantly taken place in SOPE. First, it is notable that the majority of rearranged regions in SOPE are bounded by IS elements in SOPE (fig. 2), and IS elements have been implicated previously in driving intragenomic rearrangements in other endosymbionts and obligatory intracellular pathogens (Song et al. 2010). Second, SOPE, but not strain HS, has a highly disrupted (nonpolarized) pattern of GC skew that is atypical among prokaryotic genomes (fig. 2) (Francino and Ochman 1997; Frank and Lobry 1999) (Rocha 2004). Such perturbations in GC skew are expected to arise when rearrangements occur that violate the conservation of strand-specific replicational symmetry. These perturbations can be visualized in figure 2, where the color scheme highlights rearrangements involving strand switching. In addition to perturbing GC skew, figure 2 also shows that the intragenomic rearrangements in the SOPE chromosome have disrupted the distribution of FtsK orienting polarized sequence (KOPS) motifs (Bigot et al. 2005; Levy et al. 2005). KOPS sites are short DNA sequences (GGGNAGGG) that are polarized from the replication origin to the *dif* site on the leading strands of the chromosome and serve to direct FtsK translocation of chromosomal DNA to daughter cells at the septum during chromosome replication and cell division. As is the case for GC skew, the distribution and strand bias of KOPS in strain HS is typical. It is also noteworthy that the SOPE genome has lost a portion of the terminus region of the chromosome that contains the *dif* site (which is clearly identifiable in strain HS). The *dif* site is recognized by the Xer recombination system that facilitates the resolution of concatenated chromosomes that are generated during replication (Carnoy and Roten 2009). It should be noted that SOPE shares the same morphology as *dif* mutants in *E. coli* (fig. 1), which are characterized by cells that form long filaments (Kuempel et al. 1991; Blakely et al. 1993). These mutants are unable to decatenate interlocked chromosomes resulting from recombination between chromosome copies during replication (Kuempel et al. 1991). Thus, not only is there evidence that SOPE has undergone a large number of recent genomic rearrangements but it also seems likely that these rearrangements have had a deleterious impact upon the replication system.

### Gene Duplication Events in SOPE

In addition to genome wide rearrangements, IS elements appear to have mediated partial genome duplications in SOPE. We detected a total of seven duplicated chromosomal regions comprising more than one CDS, ranging in size from 2.5 to 23.8 kb (supplementary file S1, Supplementary Material online). The most striking duplication in SOPE is 13,476 bases in length and encompasses the genes encoding the molecular chaperone GroEL and its cochaperone GroES as well as the adjacent genes *yjdC, dipZ, cutA, aspA, fxsA, yjeI, yjeK,* and *efp.* Nucleotide alignments of the whole duplicated region show that the two copies are 99.9% identical, differing by only four single base indels and 13 nucleotide substitutions, indicating that the duplication took place recently. Notably, both copies of *groEL* and *groES* maintain intact ORFs and are therefore predicted to be functional. The duplicated regions are bounded by IS256 elements, implying that an IS element-mediated recombination event catalyzed the duplication. In other mutualistic symbionts, including SOPE, *groEL* and *groES* have been shown to be expressed at very high levels to compensate for the presence of aberrant polypeptides and/or the absence of alternative repair pathways that function to rescue misfolded proteins (Ishikawa 1984; Moran 1996; Charles et al. 1997; Fares et al. 2004; Viñuelas et al. 2007; Stoll et al. 2009). Because SOPE has a very large complement of disrupted genes that are expected to yield truncated polypeptides with folding constraints, we hypothesize that the duplication of the *groEL* region facilitated an adaptive benefit. This could also be true for other genomic duplications that have taken place in SOPE, although the nature of such benefits is not immediately obvious when considering the genes involved (supplementary file S1, Supplementary Material online).

### Evolution of Ribosomal RNA Genes in the Transition to Symbiosis

Although the numbers of ribosomal RNA (rRNA) operons in bacteria can reach as many as 15 copies, it has been noted that many long-established primary endosymbionts maintain only one or two operon copies (Moran et al. 2008). The number of rRNA operons in bacteria has been shown to influence growth rate (Stevenson and Schmidt 2004) and the ability to respond quickly to nutrient availability (Klappenbach et al. 2000). Neither of these traits is expected to be of great value for insect symbionts due to the fact that they inhabit a relatively static, competition-free environment.

The genome of strain HS was found to maintain seven rRNA operons in total comprising the 16S, 23S, and 5S rRNA, with two operons maintaining an additional copy of the 5S rRNA gene. In contrast, the SOPE genome was found to maintain only two complete rRNA operons, along with three additional complete copies and a partial copy of the 16S rRNA gene. Not surprisingly, one of the complete rRNA operon copies maintains an intergenic tRNA^Glu^, whereas the other encodes tRNA^Ile^ and tRNA^Ala^, ensuring that all three rRNA operon-associated tRNAs have been retained. Although the retention of the three isolated copies of 16S rRNA is intriguing and may have some cryptic adaptive value, it is equally conceivable that it simply reflects stochastic events inherent in the process of genome degeneration. To this end, it is notable that all the rRNA genes in SOPE occupy positions in the genome that correspond contextually to the positions of the complete rRNA operons in strain HS. Thus, it appears that the isolated copies of 16S rRNA resulted from deletion events, rather than gene duplications.

Although many bacteria maintain near-identical copies of their rRNA genes as a consequence of gene conversion (Větrovský and Baldrian 2013), it was shown previously that SOPE maintains unusually divergent copies, presumably reflecting a loss of this activity (Dale et al. 2003). In addition, rRNA genes typically evolve at a very low rate, due to the fact that their sequences are highly constrained by structure and function. In a previous study, we noted that the level of sequence divergence between the 16S rRNA genes of SOPE and strain HS was unexpectedly high in comparison to the level of pairwise sequence identity observed between orthologous protein-coding genes in these bacteria (Clayton et al. 2012). Thus, we elected to further investigate the nature of mutations in the 16S rRNA genes of SOPE along with (for context), its sister species, the primary endosymbiont of the maize weevil *S. zeamais* (SZPE), and the closely related insect symbiont *So. glossinidius*. This was achieved by classifying mutations in the context of a 16S rRNA secondary structure model (Pei et al. 2010). This analysis facilitated the classification of mutations in stem regions of the 16S rRNA molecule as either structurally conservative or structurally disruptive. The results showed that both SOPE and SZPE have an unusually high ratio of disruptive to conservative mutations in their 16S rRNA genes, relative to strain HS and *So. glossinidius* (supplementary file S2, Supplementary Material online). We then performed a second analysis using the 16S rRNA variability map that was derived from a large number of bacterial species (Wuyts 2001). This analysis facilitated the classification of mutations in the entire 16S rRNA molecule according to rarity. Conspicuously, the 16S rRNA genes of SOPE and SZPE were found to maintain a high number of substitutions at sites that typically display low variability. Taken together, these results indicate that the 16S rRNA genes in SOPE and SZPE are evolving under relaxed functional constraints, despite the fact that these symbionts have a recent symbiotic origin.

### Nucleotide Substitution Rates and Prediction of Cryptic Pseudogenes

**Fig. 4.—:**
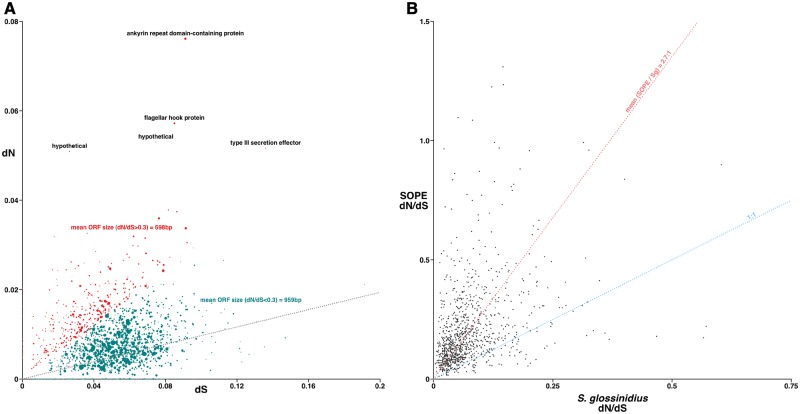
Plot of d*N* versus d*S* of orthologous genes in strain HS and SOPE. (*A*) Plot of d*N* versus d*S* of 1,601 orthologous genes in strain HS and SOPE. Each point depicted on the plot represents a single gene with the radius of each point proportional to gene size. Mean d*N*/d*S* ratio is plotted as a gray dotted line. Genes with a d*N*/d*S* ratio greater than 0.4 are annotated with their product. Genes with a d*N*/d*S* ratio greater than 0.3 are depicted in red and those genes with d*N*/d*S* ratios less than 0.3 are depicted in green. Mean ORF size was calculated for genes with d*N*/d*S* greater than 0.3 (red) and d*N*/d*S* less than 0.3 (green). (*B*) Plot of SOPE d*N*/d*S* versus *Sodalis glossinidius* d*N*/d*S* for 1,229 orthologous genes in strain HS, SOPE, and *So. glossinidius.* Each point on the plot represents a single orthologous gene.

The number of nonsynonymous substitutions per nonsynonymous site (d*N*) and the number of synonymous substitutions per synonymous site (d*S*) values were calculated for 1,602 orthologous genes in strain HS and SOPE. The graph of d*N* versus d*S* depicts each gene as an individual point, with the radius of each point proportional to ORF size (fig. 4). The gene with the highest d*N* value encodes a predicted ankyrin repeat domain protein. Ankyrin repeat domain proteins in eukaryotes have been shown to function in protein–protein interactions (Sedgwick and Smerdon 1999), and it has been hypothesized that ankyrin repeat domain proteins have a role in the cytoplasmic incompatibility generated by *Wolbachia* in its insect host (Tram and Sullivan 2002; Tram et al. 2003).

Analysis of the plot of d*N* versus d*S* revealed that genes with the highest d*N*/d*S* ratios were smaller in size. The number of genes with a d*N*/d*S* ratio ≥ 0.3 (plotted in red) (supplementary file S3, Supplementary Material online) is approximately equivalent to the number of genes predicted to be “cryptic pseudogenes” in a previous study (Clayton et al. 2012). The mean ORF size of these genes is significantly smaller than those with a d*N*/d*S* ratio less than 0.3 (plotted in green). This size difference supports the notion that the genes with a d*N*/d*S* ratio greater than 0.3 are a subset of genes evolving under relaxed selection that have not yet been disrupted by a mutation (Clayton et al. 2012). Of course due to the extremely close relationship between strain HS and SOPE, some estimates of d*N* and d*S* (especially from genes of small size) yield relatively large standard errors (supplementary file S3, Supplementary Material online). Thus, the results presented in this study should be taken with “a grain of salt” and not considered to provide a definitive inventory of cryptic pseudogenes in SOPE.

### Functional Predictions of the Protein-Coding Genes in SOPE

#### Genetic Machinery

The predicted protein-coding gene inventory of SOPE was analyzed in comparison with that of *So. glossinidius*, taking advantage that both of them appear to be unique subsets of the gene complement found in their close relative strain HS (Clayton et al. 2012) and using the abundant functional information available for the orthologous genes in *E. coli*. This analysis revealed that the essential machinery needed for the storage and processing of genetic information is well preserved in both SOPE and *So. glossinidius*, with a nearly complete set of genes needed for DNA replication, transcription, and translation. The only two genes absent in SOPE that have been considered essential for DNA replication in *E. coli* are *dnaC* and *dnaT*. However, only *dnaC* is present in *So. glossinidius*, and neither are universally present in endosymbiont genomes (Gil et al. 2004).

A general feature of endosymbionts with highly reduced genomes is the loss of DNA repair and recombination machinery. The loss of *recF*, a gene involved in DNA recombinational repair, was previously identified in SOPE and SZPE (Dale et al. 2003). The complete genome analysis revealed that other genes involved in this pathway are also pseudogenized in SOPE (*ruvA*, *ruvB,* and *recG*). Nevertheless, a minimal set of genes required for the mechanisms of base excision, nucleotide excision, and mismatch repair appear to remain intact.

In contrast with more ancient endosymbionts, SOPE has maintained a significant number of genes associated with regulatory functions, a characteristic that it shares with *So. glossinidius*, although the preserved genes are not identical. In addition to many transcriptional and posttranscriptional regulators, SOPE retains three intact sigma factors: *rpoD* (σ70, primary sigma factor during exponential growth) (Jishage et al. 1996), *rpoH* (σ32, primary sigma factor controlling the heat shock response during log-phase growth) (Grossman et al. 1984; Yura et al. 1984), and *rpoS* (alternative master regulator of the general stress response) (Maciag et al. 2011). In *So. glossinidius, rpoS* is a pseudogene, however, it retains *rpoE* (σ24, a minor sigma factor that responds to the effects of heat shock and other stresses on membrane and periplasmic proteins) (Erickson et al. 1987; Wang and Kaguni 1989; Ades et al. 2003) and *rpoN* (σ54, which controls the expression of nitrogen-related genes, also involved in the nitric oxide stress response) (Hirschman et al. 1985; Hunt and Magasanik 1985; Reitzer et al. 1987; Gardner et al. 2003), both of which have been pseudogenized in SOPE.

#### Metabolic Reconstruction

**Fig. 5.—:**
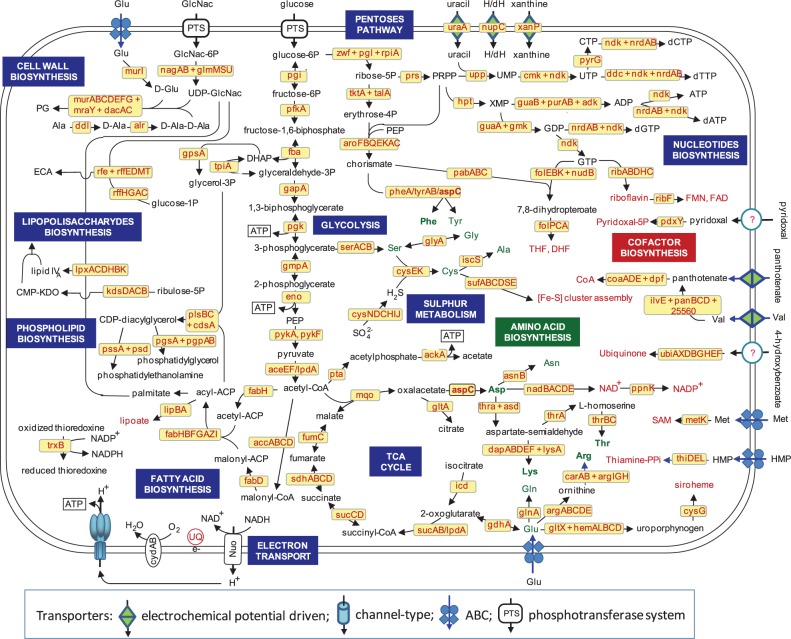
Overview of SOPE metabolism. The names in the yellow boxes indicate the genes predicted to be responsible for a given reaction. The generation of ATP is indicated. Abbreviations (besides the accepted symbols): CMP-KDO, CMP-3-deoxy-d-manno-octulosonate; DHF, dihydrofolate; ECA, enterobacterial common antigen; GlcNac-6P, N-acetyl glucosamine-6-phosphate; H/dH, nucleoside not G; DHAP, dihydroxyacetonephosphate, hydroxymethylpyrimidine; PEP, phospoenolpyruvate; PG, peptidoglycan; PRPP, phosphoribosyl pyrophosphate; SAM, S-adenosylmethionine; THF, tetrahydrofolate; UQ: ubiquinone.

The detailed analysis of the predicted metabolic capabilities of SOPE (fig. 5) indicates that it should be able to synthesize most essential amino acids. However, the genes responsible for the synthesis of tryptophan and methionine are pseudogenized, and the complete operon involved in the biosynthesis of histidine has been lost. Regarding genes involved in the biosynthesis of cofactors and vitamins, SOPE should to be able to synthesize most of them, including the complete pathways for the synthesis of riboflavin, nicotinamide adenine dinucleotide (NAD+), nicotinamide adenine dinucleotide phosphate (NADP+), coenzyme A, thiamine, and folate. The biosynthesis of lipoic acid, ubiquinone, and siroheme could also be performed by SOPE. However, it has lost the *edp* gene encoding the enzyme needed to perform the first step in the synthesis of pyridoxine. The complete pathway for biotin synthesis (excluding *bioH*) is pseudogenized.

SOPE appears to retain complete pathways for energy metabolism, as well as lipid and nucleotide biosynthesis, similar to what is found in *So. glossinidius*. However, alternative pathways for nucleotide biosynthesis appear to be disrupted. For example, the purine metabolism pathway that appears to be intact in *So. glossinidius* suffers from a pseudogenized *purH* gene, responsible for the synthesis of inosine monophosphate, although the rest of the pathway remains intact. Therefore, SOPE probably employs the same solution as *Mycoplasma genitalium*, using the enzyme hypoxanthine phosphoribosyltransferase (EC 2.4.2.8, encoded by the gene *hpt*) for the synthesis of guanosine monophosphate and adenosine monophosphate from phosphoribosyl pyrophosphate (PRPP) and guanine or adenine. This alternative pathway has been inactivated in *So. glossinidius*. Pyrimidine biosynthesis appears to be complete in *So. glossinidius*, but the pseudogenization of *udk* and *tdk* genes in SOPE likely forces the biosynthesis of cytosine and thymine nucleotides from uracil.

A previous comparative genomics study, using genome arrays hybridization (Rio et al. 2003), suggested that SOPE had retained many d-glucosidases, which can catabolize complex plant sugars. However, the availability of the whole genome revealed that most genes encoding such enzymes are pseudogenized. Nevertheless, it has retained *malP*, allowing the degradation of starch (the major constituent of rice) to obtain glucose-1-phosphate.

As in other endosymbionts, SOPE is undergoing reduction in the number and diversity of transport-associated genes. It still retains intact genes encoding ABC transporters for several cell envelope precursors including, N-acetyl-d-glucosamine, lipopolysaccharides (LPS), lipoproteins, and phospholipids. SOPE also contains an ABC transporter for hydroxymethylpyrimidine, which is needed for the synthesis of thiamine diphosphate. In contrast, *So. glossinidius*, which is unable to synthesize thiamine, lacks the transporter for a thiamine precursor hydroxymethylpyrimidine but retains a thiamine transporter. Also present in SOPE are ABC transporters for polyamines and several amino acids, such as glutamate, aspartate, and d-methionine as well as transporters for sulfate, iron complexes, and zinc. In addition to its ABC transporter, N-acetylglucosamine can also be internalized through a phosphotransferase system (PTS). N-acetylglucosamine can be used as a carbon source by *So. glossinidius* and probably by SOPE as well. The only additional PTS that has been preserved in SOPE facilitates the intake of glucose. SOPE has lost those PTSs predicted to be used for the intake of maltose, mannose, and mannitol that are preserved in *So. glossinidius*. Additionally, SOPE possesses several electrochemical potential-driven transporters for various nitrogenous bases, aromatic and branched amino acids, as well as glutamate, aspartate, gluconate, and glycerate.

#### Cell Envelope and Host–Symbiont Interactions

The comparative analysis of the genes involved in peptidoglycan (PG) biosynthesis and turnover in SOPE, *So. glossinidius,* and strain HS reveals that all three likely retain a canonical cell wall. All genes involved in the initial stages of PG biosynthesis are preserved, and only slight differences in enzymes required during the final biosynthetic stage (Scheffers and Pinho 2005) are found in the three analyzed species. All of them have retained *mrcB*, encoding penicillin-binding protein 1B (PBP1B), one of the bifunctional, inner membrane enzymes catalyzing the transglycosylation and transpeptidation of PG precursors in the formation of the murein sacculus. The gene encoding the second enzyme with this same function, penicillin-binding protein 1A (PBP1A, encoded by *mrcA*), is pseudogenized in SOPE. Additionally, in *E. coli*, there are two outer membrane lipoproteins that are critical for PBP1 function, LpoA and B, acting on MrcA and B, respectively (Paradis-Bleau et al. 2010; Typas et al. 2010). As expected, *lpoA* and *lpoB* are intact in strain HS and *So. glossinidius*, but only *lpoB* remains intact in SOPE. Although a PBP1B-PBP1A double mutation is lethal in *E. coli* (Spratt 1975; Suzuki et al. 1978; Kato et al. 1985; Wientjes and Nanninga 1991), a single functional PBP1 is sufficient for murein synthesis, since PBP1A mutants do not exhibit defects in growth or cell morphology (Spratt and Jobanputra 1977). Therefore, it appears that SOPE may not have any serious defects in PG formation, which may explain in part why it can trigger the immune response of the rice weevil when injected in the hemolymph following its isolation from the bacteriome or after heat treatment (Anselme et al. 2008; Vigneron et al. 2012).

Specific hydrolases, classified as muramidases, glucosaminidases, amidases, endopeptidases, and carboxypeptidases, are involved in breaking the covalent bonds of the existing PG sacculus, to enable the insertion of new material for cell growth and division (Scheffers and Pinho 2005). Many genes encoding proteins with these functions appear pseudogenized or are absent in SOPE and *So. glossinidius*, when compared with strain HS. Nevertheless, most organisms have redundant enzymes involved in these functions, and although many of them are still poorly characterized, some that would be essential for the maintenance of a well-structured cell wall are still present in SOPE and *So. glossinidius*.

SOPE, *So. glossinidius,* and strain HS are thought to be able to synthesize simplified LPS. All three lack enzymes to synthesize the O-antigen, and some genes involved in the modification of the core region, which have been associated with virulence (Heinrichs et al. 1998; Yethon 1998; Regué et al. 2001) are absent or pseudogenized. Although most genes encoding lipid A-modifying enzymes that have been found in *So. glossinidius* are present in SOPE, *pagP* is absent. The PagP protein mediates the palmytoylation of lipid A, a structural modification associated with bacterial resistance to alpha-helical antimicrobial peptides (AMPs) such as cecropin (Pontes et al. 2011).

All three analyzed species may be able to synthesize the enterobacterial common antigen, a family-specific surface antigen restricted to the Enterobacteriaceae that is shared by almost all members of this family, although it is not present in some endosymbionts. Its biological function remains unknown, although it has been suggested that in *Salmonella enterica,* it is associated with bile resistance (Ramos-Morales et al. 2003).

In Gram-negative bacteria, up to six different specialized systems have been described for the translocation of proteins through the inner and outer membranes. Some proteins can be directly exported in a single step through the cell wall, whereas others are first exported into the periplasm through the Sec translocation and twin-arginine translocation (Tat) pathways. Both the Sec and Tat translocation systems are present and appear to be intact in SOPE, although several genes encoding accessory subunits have been lost or appear pseudogenized.

Recent data have provided strong evidence that outer membrane proteins (OMPs) fulfill pivotal functions in host–symbiont interactions (Weiss et al. 2008; Login et al. 2011; Maltz et al. 2012). Therefore, we focus not only on the OMPs that are encoded in the SOPE and *So. glossinidius* genomes but also their ability to properly produce and place these proteins in the cell envelope. The signal transduction system EnvZ/OmpR that regulates porin expression in *E. coli* is present both in SOPE and *So. glossinidius*. The OMP assembly complex BamABCDSmpA is also complete in SOPE and *So. glossinidius*. At least one member of the AsmA family, needed for the correct assembly of OMPs, is present in each genome. However, the specific OMPs that have been preserved differ in both organisms. SOPE retains OmpC and OmpX, whereas *So. glossinidius* retains OmpF and a modified version of OmpA, which is involved in modulation of host tolerance to *So. glossinidius* (Weiss et al. 2008). *Escherichia coli* double mutants *ompF-ompC* do not survive well (Darcan et al. 2003), so it seems that at least one of them must be present, as is the case in these symbionts. Overlay experiments have shown that OmpA and OmpC are able to interact with the antimicrobial peptide Coleoptericin A (ColA) and presumably facilitate its delivery inside the bacterial cytosol. ColA was shown to alter bacterial cell division, through its interaction with GroEL, which results in SOPE gigantism and its seclusion within the bacteriome organ (Login et al. 2011). Although the function of OmpX has not been empirically determined in SOPE, it belongs to a family of highly conserved proteins that appear to be important for virulence by neutralizing host defense mechanisms (Heffernan et al. 1994). It has been proposed to function in cell adhesion and invasion, as well as in the inhibition of the complement system (Vogt and Schulz 1999).

**Fig. 6.—:**
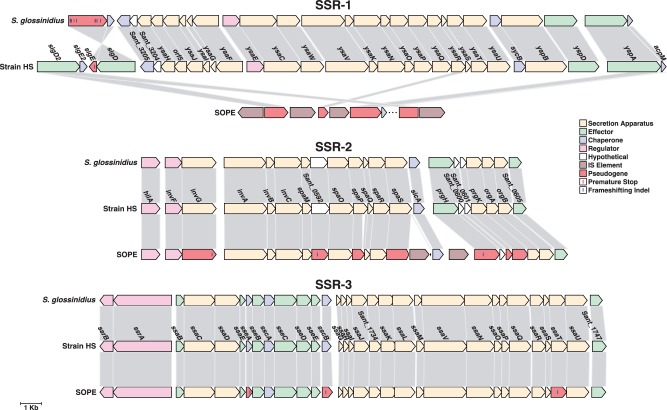
Comparison of TTSS gene clusters*.* Scalar illustration of the chromosomal regions encoding SSRs (SSR1–3) in *Sodalis glossinidius* (top), strain HS (middle), and SOPE (bottom). Genes are colored according to their predicted functions as secretion apparatus (tan), secreted effectors (green), chaperones (purple), transcriptional regulators (pink), hypothetical (white), or IS elements (brown). Pseudogenes are colored in red. Frameshifting indels and premature stops are shown as blue and red tick marks at their respective positions within the ORF. Pseudogenes without tick marks are inactivated by an IS element insertion within an ORF or by truncations of greater than 10% compared with the intact strain HS ortholog. Gene names are shown based on the strain HS annotation.

TTSSs have been preserved in several insect endosymbionts, where they are postulated to be involved in the invasion of the host cells (Hueck 1998). To further understand the gene content and organization of the TTSSs or *Sodalis* symbiosis regions (SSR) (Dale et al. 2005) in SOPE, we analyzed and compared the gene content of these three distinct chromosomal regions, SSR-1, SSR-2, and SSR-3, in strain HS, *So. glossinidius,* and SOPE (fig. 6). The SSR-2 and SSR-3 islands of SOPE share a high level of conservation both with *So. glossinidius* and strain HS. SSR-2 is most closely related to the SPI-1 pathogenicity island found in *Sa. enterica* and may play a role in intracellular protein secretion in *So. glossinidius* (Dale et al. 2005), whereas SSR-3 is most similar to the SPI-2 pathogenicity island found in *Sa. enterica* where it plays an important role in virulence (Figueira and Holden 2012). However, our analysis indicates that many of the TTSS genes have been inactivated or deleted in SOPE. SSR-1, which is most closely related to the *ysa* pathogenicity island found in *Yersinia enterocolitica* and has been shown to play a role in host cell entry in *So. glossinidius* (Dale et al. 2005)*,* is the most extensively degraded in SOPE. Of the four genes remaining in the SSR-1 island of SOPE, only one appears to be intact and potentially functional, whereas the others have been inactivated by IS element insertions.

#### Environmental Information Processing

In bacteria, extracellular signals are transduced into the cell predominantly by two-component systems (TCSs), allowing them to sense and adapt to environmental changes (Mitrophanov and Groisman 2008). Typically, a TCS consists of a sensor kinase, which responds to specific signals by modifying the phosphorylation state of an associated response regulator (Gao et al. 2007). There are a variety of functions that can be controlled through TCSs, including nutrient acquisition, energy metabolism, adaptation to physical or chemical aspects of the environment, and even virulence. Therefore, it is not surprising that these elements are some of the first to be lost soon after the onset of a stable intracellular symbiosis. Thus, although traces of several TCSs can be found in SOPE and *So. glossinidius*, one or both components often harbor mutations. Nevertheless, several two-component pairs still appear intact, some of which might be relevant for the host–bacterial association. In addition to the aforementioned EnvZ/OmpR TCS, SOPE has retained the PhoP/PhoQ system, whose functions include the control of TTSS gene expression, AMP resistance, and modification of the lipid A portion of the LPS through regulation of the *arn* operon in *So. glossinidius* (Pontes et al. 2011).

#### Description of *Candidatus* Sodalis pierantonius str. SOPE

Previous phylogenetic analysis indicated that SOPE belongs to the *Sodalis*-allied clade of insect symbionts, sharing more than 97% sequence identity in their 16S rDNA sequences (Heddi et al. 1998; Charles et al. 2001; Clayton et al. 2012). Therefore, we propose that SOPE should be included in the *Sodalis* genus. Following Murray and Stackebrandt (1995), microorganisms partially characterized and not cultivated on laboratory media might be given the designation “*Candidatus*.” Consequently, we propose to name the lineage belonging to the cereal weevils *Sitophilus* spp. Endosymbionts as *Candidatus* Sodalis pierantonius str. SOPE. This species name refers to the Italian zoologist Umberto Pierantòni (1876–1959), who first described the symbiosis in *Sitophilus* spp. weevils (Pierantoni 1927). The description of *Candidatus* Sodalis pierantonius str. SOPE is as follows: phylogenetic position, γ3-subclass of Proteobacteria; cultivation, not cultivated on cell-free media; Gram reaction, negative; morphology, pleiomorphic rods, from 3–4 to 100 μm in length, 1–2 μm in diameter, surrounded by a mucopolysaccharide-like substance; basis of assignment, 16S rDNA sequences and genome analysis; association and host, intracellular symbionts of the cereal weevils *Sitophilus* spp. (described in *S. oryzae*, *S. zeamais,* and *S. granarius*).

## Discussion

This study focuses on the complete annotation and comparative analyses of the genomes of the rice weevil primary endosymbiont (known as SOPE), now designated *Candidatus* Sodalis pierantonius str. SOPE and the recently described closely related, free-living strain HS. Although many insect symbionts have now been completely sequenced, SOPE is unique because of its very recent symbiotic origin (Lefèvre et al. 2004; Clayton et al. 2012). In terms of the age of association, SOPE is akin to many facultative “secondary” symbionts that have relatively large genome sizes and reside in multiple host tissues alongside a “primary” (ancient) nutritional symbiont. Yet, SOPE resides alone in a specialized structure (bacteriome) just like many obligate “primary” symbionts that have small genome sizes and long established associations with their insect hosts. Furthermore, similar to those “primary” symbionts, SOPE has a substantial beneficial effect on the basic physiological fitness of its weevil host. In the laboratory, weevils lacking SOPE display substantially reduced fecundity and flight ability, along with a markedly increased generation time (Heddi et al. 1999). These deficiencies can be partially compensated by the addition of certain B vitamins to the insect diet (Wicker 1983), indicating that SOPE has a nutritional role in its host insect. Although one might argue that the ability to maintain aposymbiotic insects in the laboratory indicates that the relationship between the rice weevil and SOPE is not strictly obligate, it seems unlikely that aposymbiotic insects could persist in the wild given the fitness deficit associated with the loss of their symbionts. So how did the symbiosis between SOPE and its insect host achieve such a high level of integration and dependency over such a brief period of association? The answer likely lies in the finding that SOPE replaced a more ancient endosymbiont (*Candidatus* Nardonella spp.) in the weevil family Dryophthoridae (Lefèvre et al. 2004; Conord et al. 2008). Thus, SOPE is predicted to have taken over a residence that was already well honed for a bacterial symbiont, facilitating rapid adaptation toward host association and mutualism.

Although the genomes of many “primary” insect symbionts are highly reduced and gene dense, the genome of SOPE is large in size and contains a high proportion of pseudogenes and mobile genetic elements, consistent with the notion of a recent symbiotic origin (Moran and Plague 2004; Gil Garcia et al. 2008; Plague et al. 2008). Indeed, the sequencing of SOPE is arguably the most technically challenging bacterial genome sequencing and annotation project completed to date, complicated by the large amount of repetitive DNA (828,763 bases total) and the fact that many IS elements are clustered in the SOPE chromosome, mandating a transposon-mediated approach to resolve their sequences (Clayton et al. 2012).

Because of the high level of nucleotide sequence identity between SOPE and strain HS, we were able to use the genome sequence of strain HS as a “Rosetta Stone” to identify and annotate pseudogenes in SOPE. Our work shows that even at such an early stage in the evolution of a symbiotic association, there has been extensive genome degeneration characterized by gene inactivation, deletion, and IS element-mediated genome rearrangements. Our work also provides evidence of elevated rates of rRNA and protein sequence evolution. In addition, as an indicator of the extent of recent genomic perturbations, the SOPE chromosome was found to lack a characteristic pattern of GC skew that is typical of circular bacterial chromosomes (Lobry 1996; Rocha 2004). Recent intragenomic rearrangements have also disturbed the distribution of polar KOPS sites that play an important role in chromosome segregation (Bigot et al. 2005; Sivanathan et al. 2009).

Perhaps the most striking feature of the SOPE genome is the presence of massive numbers of IS elements. High numbers of IS elements have been observed in several host-restricted pathogenic enteric bacteria, such as *Shigella flexneri* strain 2457T, which has 284 total IS elements (Wei et al. 2003) and *Shigella flexneri* 2a with 314 (Jin et al. 2002), and *Orientia tsutsugamushi* strain Ikeda, which has 621 copies belonging to five different IS families (Nakayama et al. 2008). Because IS element expansions seem to be common in bacteria undergoing lifestyle transitions, it has been suggested that their effects are relatively neutral with respect to selection. This is largely due to the fact that bacteria that have recently undergone a lifestyle switch (like SOPE) often harbor a large number of dispensable genes evolving under relaxed selection. This provides an opportunity for IS elements to expand their range into a large area of neutral space. In addition, bacteria that become host restricted are anticipated to experience a reduction in effective population size that reduces the strength of natural selection, allowing more transposition events to become fixed in the population by genetic drift (Parkhill et al. 2003). Deleterious mutations fixed by genetic drift as a consequence of a reduction in the effect of natural selection can also inactivate host genes that negatively regulate IS element transposition activities (Roberts et al. 1985; Valle et al. 2007). In addition to these neutral explanations, it has also been proposed that intragenomic IS element proliferations can have adaptive consequences. For example, many IS elements maintain strong promoters that have the capability to drive the expression of exogenous genes if they are inserted into promoter regions (Reimmann et al. 1989; Schnetz and Rak 1992; Craig et al. 2002). Furthermore, IS elements can catalyze intragenomic rearrangements, as observed extensively in SOPE, leading to gene duplication events and reassortment of regulons (Mahillon and Chandler 1998; Chain et al. 2004; Nierman et al. 2004). Finally, IS elements have the potential to mediate genome streamlining, by accelerating the rate at which dispensable regions of the genome are deleted via deletogenic rearrangements (Mahillon and Chandler 1998; Fang et al. 1999; Gil et al. 2010). It is also conspicuous that the IS elements of SOPE are preferentially located in intergenic sequences, rather than within the substantial array of pseudogenes that are evolving under relaxed selection (Clayton et al. 2012). We previously suggested that the propensity of IS elements to occupy intergenic sequences might be a consequence of a mechanistic bias that prevents IS elements from interrupting essential genes (Clayton et al. 2012). However, it is also possible that they have played a role in modulating gene expression and/or silencing the expression of pseudogenes in SOPE. In addition, they have catalyzed the duplication of several chromosomal regions, including that encoding *groEL* and *groES,* and have likely mediated numerous deletogenic rearrangements in the SOPE genome.

The predicted functional analysis of the gene complement found in the SOPE genome and its comparison with that of *So. glossinidius* confirmed that, although both symbionts have undergone specific and independent gene losses compared with their close relative strain HS, the subset of genes preserved and lost in both SOPE and *So. glossinidius* are quite similar. This is an indication that the reductive process occurring in these insect-associated bacteria responds to general constraints imposed by their lifestyle (Clayton et al. 2012).

Many highly reduced endosymbiont genomes analyzed to date have lost most of their DNA repair and recombination mechanisms. It has been proposed that the accumulation of mutations in genes belonging to this category, which can be considered beneficial but not essential, might occur at the onset of the endosymbiotic relationship. This would further increase the mutational pressure on nonessential genes and reduce the possibility of genetic exchange and gene conversion through homologous recombination, making any gene loss irreversible (Moya et al. 2008). However, we found that, although genes involved in DNA recombination have been lost, the DNA repair machinery appears to be intact in SOPE.

RNA metabolism is the most evolutionarily conserved pathway in modern cells, even in endosymbionts with highly reduced genomes, and SOPE is no exception. However, the maintenance of many regulatory genes might indicate that, contrary to other long established endosymbionts, SOPE and its close relative *So. glossinidius* are still able to sense and respond to environmental signals. Although, it is not clear whether these regulatory genes play an important role in environmental sensing or if they simply exist as a stopgap to drive expression of essential genes (Pontes et al. 2011).

Our functional metabolic predictions partially confirm the results of previous physiological studies performed on symbiotic and aposymbiotic weevils (Heddi et al. 1999) indicating that SOPE is likely able to synthesize most amino acids, except for tryptophan, methionine, and histidine. Regarding vitamin provision, our predictions support the experimental results obtained by (Wicker 1983) regarding the capability of SOPE to provide riboflavin, NAD+, NADP+, and coenzyme A. In that previous work, it was also suggested that SOPE was able to provide pyridoxine, although not enough to maintain development. This fits with the observation that SOPE lacks *edp*, a gene involved in the first step of the pathway. However, we also found that SOPE appears to have retained the complete pathways to synthesize thiamine and folate, even though Wicker (1983) observed that thiamine deficient diets were not able to properly sustain symbiotic or aposymbiotic insects and that both require an external source of folate. Finally, although Wicker’s observations indicated that the lack of biotin affects fecundity only in aposymbiotic insects, the biosynthetic pathway is impaired due to the pseudogenization of all genes involved, with the exception of *bioH*. It is interesting to note this is also the only gene in biotin biosynthesis preserved in *Buchnera aphicidola* BCc, the primary endosymbiont of the cedar aphid (Pérez-Brocal et al. 2006). Furthermore, this gene appears to be nonessential for the synthesis of biotin (Rodionov et al. 2002), and additional enzymatic activities have been proposed (Sanishvili et al. 2003), which might indicate that this gene performs another essential yet uncharacterized function in endosymbionts.

The provisioning of amino acids and vitamins in SOPE and *So. glossinidius* is highly divergent. Two factors can explain this difference. First, their hosts have evolved to survive on very different diets; *S. weevils* feed on cereal kernels and *Glossina* spp. on vertebrate blood. The highly specialized diets of the insect hosts have been proposed to be responsible for the observed changes in genes involved in complex plant carbohydrates and lipid metabolism as carbon and energy sources in SOPE and *So. glossinidius* (Rio et al. 2003). Second, tsetse flies also harbor a bacteriome-associated primary endosymbiont, *Wigglesworthia glossinidia*, which is predicted to cooperate with *So. glossinidius* for the synthesis of vitamins and cofactors (Akman et al. 2002; Belda et al. 2010).

The establishment of any intracellular mutualistic symbiosis between a bacterium and a eukaryotic host implies that both organisms coevolve to adapt to the association. The bacterium develops mechanisms to overcome the physical, cellular, and immune barriers presented by the host to invade and replicate in host cells and achieve transmission to offspring. On the other hand, the host differentiates specialized cells to harbor the bacterium (Heddi et al. 1998; Braendle et al. 2003) and develops mechanisms to confine the symbiont and control its population. This can be accomplished either by controlling the nutrient provision to the bacterium (International Aphid Genomics Consortium 2010) or by mounting adapted local immune response within the bacteriocyte cells (Anselme et al. 2008; Login et al. 2011). Microbe-associated molecular patterns (MAMPs), such as PG and LPS, are capable of activating a constant host immune response through interaction with host pattern recognition receptors. Simultaneously, toxin proteins can be delivered into the host cell through secretion systems (SS) to inhibit immune response and to ensure tissue infection. Remarkably, most genes encoding PG, LPS, SS, and toxins were shown to be absent from the genomes of most long lasting insect endosymbionts. The loss of these immune eliciting elements indicates that evolutionary constraints also remove genes involved in immune signaling and attests that the modification of MAMP structure are among the adaptive functions in host–symbiont interactions (McCutcheon and Moran 2011). As SOPE retains a well-structured cell wall and activates host AMPs when injected into insect hemolymph, future studies will help to understand how SOPE manages to escape host immune effectors and how bacteriocyte local response is modulated to maintain SOPE while controlling its growth and multiplication (Anselme et al. 2008; Vigneron et al. 2012). Hence, investigation of SOPE association with weevils would provide insights into how symbionts are tolerated by the host immune system in the early steps of symbiosis and will shed light on the evolution of host-bacterial signaling in parallel with PG gene deletions and MAMP structure modification.

Many pathogenic bacteria possess TTSSs, encoded within specialized genomic islands that facilitate interactions with host cells. TTSSs have now been identified in many insect endosymbionts, where they have been shown to play a role in host cell invasion (Dale et al. 2001, 2005). The structure of the symbiotic islands encoding TTSS in SOPE is intriguing (fig. 6). SSR-1, which has been shown to be involved in host cell invasion in *So. glossinidius* (Dale et al. 2005), has almost been erased from the SOPE genome. An alternative hypothesis suggests that a simplified flagellar apparatus might be occupying this function (Young et al. 1999; Maezawa et al. 2006). Both flagellar apparatuses are partially degraded in SOPE. Even though some genes involved in flagellar synthesis are duplicated, many of them are pseudogenized.

On the other hand, the SSR-2 and SSR-3 islands, which have been related with intracellular protein secretion in *So. glossinidius* and virulence in *Sa. enterica*, are quite well preserved. Notably, although the structural elements of the apparatus as well as most chaperones and regulators are highly conserved among SOPE, strain HS, and *So. glossinidius*, many predicted effector proteins have been disrupted or lost in each symbiont. These proteins might function as virulence factors in strain HS, a function that might have been abandoned in the insect-associated symbionts. Overall, the differential loss of TTSS-encoding components observed in *So. glossinidius*, *S. melophagi* (Chrudimský et al. 2012), and SOPE might be an indication that the mandate for TTSS functions varies according to the context of the symbiosis. Nevertheless, the presence of all three symbiotic regions in strain HS and the high degree of conservation between strain HS and *So. glossinidius* indicates that these TTSS-encoding regions are of ancestral origin to these *Sodalis*-allied symbionts and have not been acquired independently by lateral gene transfer. It is also consistent with the notion that the functions of all three islands have been retained in *So. glossinidius*.

In bacteria, pairwise estimates of d*N*/d*S* typically fall within the range of 0.04–0.2 for functional genes that are evolving under stabilizing selection, whereas genic sequences that have been rendered inactive (pseudogenes) are expected to have d*N*/d*S* ratios that approach parity (Rocha et al. 2006). In this study, we found that the number of genes having d*N*/d*S* ratios greater than 0.3 is approximately equal to the number of “cryptic pseudogenes” in SOPE estimated in a previous study (Clayton et al. 2012) and have an average size that is significantly smaller than the average size of genes with d*N*/d*S* ratios less than 0.3. This is consistent with the notion that this subset of genes is evolving under relaxed selection. Moreover, our results demonstrate that both protein-coding genes and rRNAs are evolving at a higher rate in SOPE relative to *So. glossinidius* (fig. 4), indicating that SOPE, as a primary nutritional symbiont, is degenerating more rapidly than *So. glossinidius*.

This work provides insight into the early stages of genome degeneration in a recently derived insect primary endosymbiont. Our work shows that SOPE has undergone very rapid genome degeneration concomitant with the onset of host association. The high rate of degeneration may be due to SOPE replacing a more ancient symbiont and moving into a niche that was already well crafted for habitation by a symbiont with a small genome, facilitating an immediate relaxation of selection on many genes in the ancestral SOPE gene inventory. An extensive IS element expansion in SOPE appears to have catalyzed duplications of several chromosomal loci including a region encoding *groEL* and *groES*. The duplication of these genes likely has an adaptive benefit, assisting in the folding of proteins whose sequences have been compromised by deleterious mutations in the process of genome degeneration. The IS element expansion has also mediated numerous genome rearrangements and deletions that might also be beneficial in nature. The forces shaping the evolution of the bacterial genome are clearly very potent in the nascent stages of symbiosis and are expected to facilitate rapid specialization of the symbiont gene inventory toward its given insect host.

## Supplementary Material

Supplementary files S1–S3 are available at *Genome Biology and Evolution* online (http://www.gbe.oxfordjournals.org/).

Supplementary Data
