# Supplementary material for: Genome Degeneration and Adaptation in a Nascent Stage of Symbiosis
Source: Genome Biol Evol. 2014 Jan 8;6(1):76–93. doi: 10.1093/gbe/evt210 (PMC3914690; doi:10.1093/gbe/evt210)
Supplement: Supplementary Data [file supp_6_1_76__index.html]

Genome Degeneration and Adaptation in a Nascent Stage of Symbiosis — Genome Degeneration and Adaptation in a Nascent Stage of Symbiosis — Supplementary Data 

# Genome Degeneration and Adaptation in a Nascent Stage of Symbiosis

## Supplementary Data

files

**Files in this Data Supplement:**

- Supplementary Data - xls file
- Supplementary Data - jpg file
- Supplementary Data - xls file
