# Supplementary figures and images for: Genome Degeneration and Adaptation in a Nascent Stage of Symbiosis
Source: Genome Biol Evol. 2014 Jan 8;6(1):76–93. doi: 10.1093/gbe/evt210 (PMC3914690; doi:10.1093/gbe/evt210)

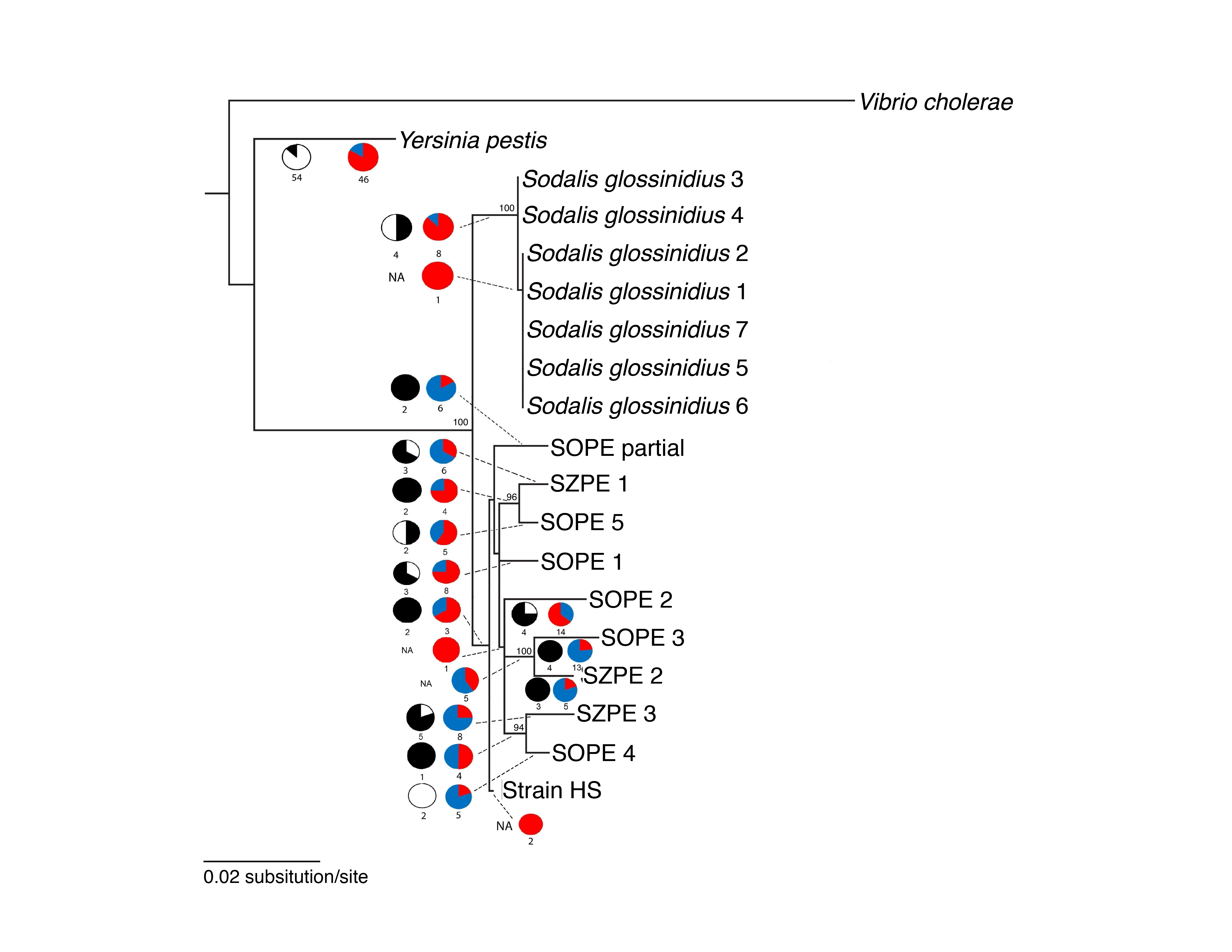

Supplement: Supplementary Data [file supp_evt210_Additional_file_2_withbootstrap.jpg]
